# Supplementary material for: Identification of photoperiod-induced specific miRNAs in the adrenal glands of Sunite sheep (Ovis aries)
Source: Front Vet Sci. 2022 Jul 22;9:888207. doi: 10.3389/fvets.2022.888207 (PMC9354845; doi:10.3389/fvets.2022.888207)
Supplement: Supplementary file 1 [file Data_Sheet_1.ZIP › Supplementary file/miRDeep2 file.zip!/miRDeep2 file/Know_Structure/SP42A3/oar-mir-107.pdf]

miRBase precursor : oar-mir-107
Total read count : 5573
oar-miR-107 read count : 5565
remaining reads : 8

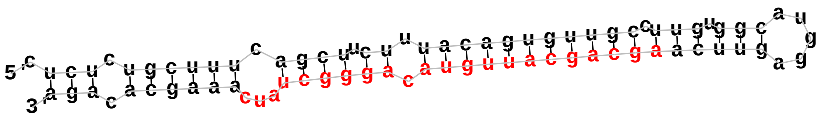

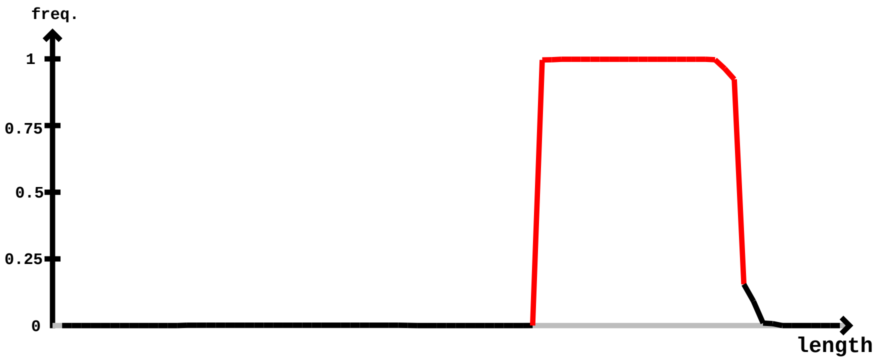

oar-miR-107

```
5'- cucucugcuuucagcuucuuuacaguguugccuuguggcauggaguucaagcagcauuguacagggcuaucaaagcacaga   -3'    exp
    .(((.((((((.((((.((.(((((((((((.(((.(((.....))))))))))))))))).))))))...)))))).)))   reads      mm        sample
    ............agcuucuuuacagugCugccuug..............................................   3          1         seq
    ............agcuucuuuacagugCugccuugu.............................................   4          1         seq
    ..............cuucuuuacagugCugccuug..............................................   1          1         seq
    ................................................aagcagcauuguacagggcuau...........   1          0         seq
    .................................................agcagcauuguacagggc..............   10         0         seq
    .................................................agcagcauuguacagggcu.............   185        0         seq
    .................................................agcagcauuguacagggcua............   224        0         seq
    .................................................agcagcauuguacagggcuau...........   4273       0         seq
    .................................................agcagcauuguacagggcuauc..........   353        0         seq
    .................................................agcagcauuguacagggcuauca.........   456        0         seq
    .................................................agcagcauuguacagggcuaucaa........   12         0         seq
    .................................................agcagcauuguacagggcuauGaaa.......   37         1         seq
    ..................................................gcagcauuguacagggcuau...........   4          0         seq
    ...................................................cagcauuguacagggcuau...........   6          0         seq
    ...................................................cagcauuguacagggcuauca.........   1          0         seq
    ...................................................cagcauuguacagggcuauGaaa.......   3          1         seq
```
